# An Illustrated System of Human Anatomy, Special, General, and Microscopic

**Published:** 1849-04

**Authors:** 


Art. V. — *An Illustrated System of Human Anatomy, Special, General, and Microscopic.* By Samuel George Morton, M. D., Penn. and Edinb., Member of the Medical Societies of Philadelphia, New York, Boston, Edinburgh, and Stockholm; author of 'Crania Americana,' 'Crania Egyptica,' etc. With three hundred and ninety-one engravings on wood. "*Oculis subjecta fidelibus.*" Philadelphia: Grigg, Elliot & Co. 1849. pp. 642. Royal 8vo.

We like to see new works on Anatomy. Each new one adds another to the means of study. They are not all equally good, some having one excellence and some another, but all contain something valuable, and we could wish them to be as common as spelling-books, or office-seekers, or heroes in the Mexican war.

All concede the necessity of a knowledge of anatomy and physiology to the medical man, but all do not agree as to the amount deemed necessary. While some are satisfied with a very superficial knowledge, others demand that it shall be profound, intimate, minute. We count ourselves among the latter. We think that the professional man cannot know too much of these matters, since they are not merely the ground-work of all his subsequent studies, but they cement, bind, and harmonize all his acquisitions in other departments. To be sure there are those who have the reputation of being pretty good doctors, and who think themselves pretty good doctors, who can scarcely tell the difference between a muscle and a mucous membrane, or between the basilar artery and the basilic vein. They have some general notions, such as that the liver is below the diaphragm and secretes bile, whence comes 'biliousness;' that the stomach is somewhere near the end of the breastbone, and furnishes what is called gastric juice, of great use in digestion; that the heart is situated somewhere in the chest, and sends blood throughout the body, the said blood being occasionally "full of humors." But of any knowledge of the structure of the liver, of the situation of the cardia and pylorus, of the uses of the mitral and semilunar valves, they are as innocent as is a babe unborn. They are practical men, forsooth; they have in their minds a close association between calomel and salivation, castor oil and cathartics, Dover's powder and diaphoretics, ipecac. and emetics. The "disgusting" details of anatomy, the abstruse mysteries of physiology, are not for them; they leave these to the plodding theoretical fellows, who have nothing else to do. But they place great value on anatomical plates, and dry preparations of the muscles, nerves, and bloodvessels, and are delighted when "lectures on a mannikin" are advertised by some itinerant quack whose ignorance is equalled by only two things in the world, viz: his impudence and the credulity of the public.

The picture is not overcharged, and the originals are too often to be seen. But such are rapidly passing away and giving place to a better class.

At least we hope so. That which lies, it seems to us, at the bottom of all medical reform in this country is a longer, closer, and more thorough study of anatomy and physiology. Beautiful as each of them is, and attractive beyond almost anything else in the whole circle of human knowledge, students of medicine, with that fatal impatience said to be characteristic of American youth, too often hurry over them as things which are of no importance and which serve no purpose save that of impeding their progress. It is difficult to imagine how a man can do so, who keeps in view the object of his studies, to cure the ills that the body is heir to. It is no less difficult to imagine how one can look on the human body and not feel an irresistible impulse to spend his life in the study of its wonderful structure and in the development of its laws. And it seems to us that he who has not this feeling should not think of fitting himself for the duties of the physician. We would make this a test, and say to him who has no love for anatomy, who does not feel an enthusiasm for it, who does not experience in it an ever new delight, to go his ways, delve the earth, seek for gold in California, pick up rags in the street, break stones on a highway, but never think of studying medicine.

Therefore it is that we hail every new work on anatomy, and welcome every new author in the field. We care not to what extent the means of study are multiplied. Each new book shows that there is one man more who acknowledges the obligations that all ought to feel—to endeavor not to leave science where he found it, but to advance it, to enlarge its boundaries, to add to its possessions. Such should be the spirit of every member of the profession, and he who enters it with other views enters where he has no business, and deserves little fellowship.

Some such feeling as this Dr. Morton confesses to have been one of the incentives to the present publication—"the wish to be enrolled among the expositors of a science which has occupied some of the best years of his life."

The work in all its externals is one of the most elegant that has ever issued from the American press, and gives a very favorable impression of the state of the arts of printing and engraving in this country. It contains a good outline of anatomy in its present state, beginning with a brief but clear exposition of the cell-doctrine. The latter is almost entirely neglected in the common elementary works, much to the annoyance and perplexity of the student. Although we are not prepared to assent to everything introduced by the cultivators of microscopic anatomy, yet as its language has invaded every department of the profession, even that of practical medicine, it is absolutely necessary that the student should early form an acquaintance with it. Dr. Morton mingles a little physiology to relieve the mere anatomical details, connecting thus the structure with the more important functions. The physiological particulars do not amount to more than hints, which the reader is to follow up elsewhere; to have made them more would have swelled the book to an inordinate size. The anatomical descriptions are concise and clear, and, aided by the numerous illustrations, make it an excellent text-book.

Notwithstanding its general excellence, the volume bears some marks of haste or of negligence which we do not like to see. Four pages are intercalated, from page 104 to page 105; the description of the patella is not in its proper place; and the hyoid bone comes in at the end of the volume like an afterthought. There is likewise a looseness of expression reprehensible anywhere, but especially in a book intended for learners whose descriptive style and phraseology may be formed after it. Thus we find such expressions as "tarsus cartilage"—"tonsil gland"—which are about as elegant as would be *femur bone*, *aorta artery*, and the like. Then the phrase, "subjected to the action of the microscope," seems to be quite a favorite with Dr. Morton, but it is certainly a very vile phrase. Want of taste, too, is shown in the mingling of Latin and English words in speaking of different structures. We hold to the canon that anatomical language should be all English or all Latin, and that such expressions as "female *genitalia*" are out of the question. Want of careful revision is shown in various places: take the following from page 599:

"Viewed from within, the cornea is exactly circular; but seen from the outside, its transverse diameter is observed to be a little less than the vertical, owing to the sclerotica overlapping it much more above and below than at the sides."

Or this from one of the notes, page 607:

"We may observe, that Drs. Todd and Bowman separate the retina into three laminæ independently of the tunica Jacobi; viz: a granulated layer externally, a vesicular gray layer in the center, and a fibrous gray layer externally."

These are matters observed in a somewhat hasty examination, and we are not disposed to multiply the in stances, or to dwell on them. In a second edition, which we trust will soon be called for, these blemishes will no doubt be removed.

The book is for sale by Peters & Webb, Main street.

